# Diversity of Activities, Emotions, and Pleasant Events and Their Associations With Mental and Cognitive Health

**DOI:** 10.1093/geroni/igab046.064

**Published:** 2021-12-17

**Authors:** Soomi Lee, Emily Urban-Wojcik, David Almeida

## Abstract

The COVID-19 pandemic dramatically changed the structure of our daily lives. One of the most significant changes is a limited opportunity to engage in face-to-face social interactions and enjoy diverse daily activities. This raises a public health concern, because diverse experiences are critical sources of health by increasing social integration, cognitive reserve, and psychological resources. Recently, two lines of research have consistently shown that activity diversity or emodiversity is associated with multiple health outcomes. However, still more integrated efforts are needed to better understand diversity of daily experiences in various aspects and their contributions to health. This symposium brings together different endeavors towards understanding how diversity of daily experiences – activity diversity, emodiversity, and variety in positive experiences – are associated with health and well-being across adulthood. The topic of this symposium is timely to discuss potential prevention approaches to protect population well-being as the pandemic evolves. Paper 1 examines activity diversity (breadth and evenness of daily activity participation) and how it is related to positive and negative emodiversity (rich and balanced emotional experiences) differently by age groups. Paper 2 investigates the longitudinal relationship between activity variety across cognitive, physical, and social domains and cognitive functioning. Paper 3 examines variety in pleasant events and its associations with mental health outcomes. Paper 4 examines whether and how negative emodiversity is associated with mental illness during COVID-19. The discussant, Dr. David Almeida will integrate key findings from these studies, discuss their theoretical and methodological contributions, and consider opportunities for future research.

